# *Reynoutria sachalinensis* extract elicits SA-dependent defense responses in courgette genotypes against powdery mildew caused by *Podosphaera xanthii*

**DOI:** 10.1038/s41598-020-60148-6

**Published:** 2020-02-25

**Authors:** Theoni Margaritopoulou, Eleftheria Toufexi, Dimosthenis Kizis, George Balayiannis, Christos Anagnostopoulos, Andreas Theocharis, Leonidas Rempelos, Yerasimos Troyanos, Carlo Leifert, Emilia Markellou

**Affiliations:** 1 Benaki Phytopathological Institute, Department of Phytopathology, Laboratory of Mycology, 8, St. Delta str., 145 61 Kifissia, Athens Greece; 2 Newcastle University, Nafferton Ecological Farming Group, School of Agriculture Food and Rural Development, Newcastle upon Tyne, NE1 7RU UK; 3 Benaki Phytopathological Institute, Department of Pesticides Control & Phytopharmacy, Laboratory of Chemical Control of Pesticides, 8, St. Delta str., 145 61 Kifissia, Athens Greece; 4 Benaki Phytopathological Institute, Department of Pesticides Control & Phytopharmacy, Laboratory of Pesticide Residues, 8, St. Delta str., 145 61 Kifissia, Athens Greece; 5 Benaki Phytopathological Institute, Department of Phytopathology, Laboratory of Non-Parasitic Diseases, 8, St. Delta str., 145 61 Kifissia, Athens Greece; 6 Centre for Organics Research, Southern Cross University, Military Rd., Lismore, NSW Australia; 7 Department of Nutrition, Institute of Basic Medical Sciences, University of Oslo, Sognsvannsveien 9, Domus Medica 0372 Oslo, Norway

**Keywords:** Plant physiology, Biotic

## Abstract

Powdery mildew (PM) caused by *Podosphaera xanthii* is one of the most important courgette diseases with high yield losses and is currently controlled by fungicides and sulphur applications in conventional and organic production. Plant derived elicitors/inducers of resistance are natural compounds that induce resistance to pathogen attack and promote a faster and/or more robust activation of plant defense responses. Giant knotweed (*Reynoutria sachalinensis*, RS) extract is a known elicitor of plant defenses but its mode of action remains elusive. The aim of this study was to investigate the mechanisms of foliar RS applications and how these affect PM severity and crop performance when used alone or in combination with genetic resistance. RS foliar treatments significantly reduced conidial germination and PM severity on both an intermediate resistance (IR) and a susceptible (S) genotype. RS application triggered plant defense responses, which induced the formation of callose papillae, hydrogen peroxide accumulation and the Salicylic acid (SA) - dependent pathway. Increased SA production was detected along with increased *p*-coumaric and caffeic acid concentrations. These findings clearly indicate that RS elicits plant defenses notably as a consequence of SA pathway induction.

## Introduction

Plants upon attack of pathogenic micro-organisms activate complex immune networks to prevent or minimize colonization of their internal structures and deprivation of nutrients. Overall, plant resistance to pathogens involves both constitutive mechanisms, which include natural physical barriers such as enforced cell walls and wax depositions on cuticle cells, and induced resistance mechanisms, which are activated within minutes to a few hours (h) after pathogen attack. Induced resistance can be demonstrated either locally (e.g. structural barriers resulting from callose deposition, cell wall lignification and necrotic lesions associated with a hypersensitive response, induction of expression of pathogenesis-related proteins and production of various end-products and antimicrobial metabolites such as phytoalexins), or systemically throughout the plant, by activation of various components of the local response *via* plant growth regulation mediated biochemical signaling pathways^1^. Apart from microbial infection, plant resistance mechanisms may also be induced by a range of elicitors which are signal-inducing compounds or agents that trigger the innate immune system and prime and/or induce defense responses^2,3^.

SA is a phenolic plant hormone that plays central role in plant defense against biotrophic pathogens^4^. This natural defense phytohormone serves as an endogenous signal to activate certain immune responses and to establish disease resistance. SA plays pivotal role in defense signaling because it triggers not only direct activation of defense responses but also activation of systemic responses after priming, as in the case of elicitor recognition^4^. The key regulator of SA signaling, transcription co-activator *NONEXPRESSER OF PR GENES 1* (*NPR1)*, interacts with transcription factors to induce the expression of anti-microbial *PR* genes^5^ and recently is considered a bona fide SA receptor^6^. *PATHOGENESIS RELATED GENE 1* (*PR1)* is the most abundant anti-microbial protein produced during defense responses and is upregulated in SA–dependent defense responses^7^.

Except SA, which typically activates defense responses against biotrophic pathogens, there are also other phytohormones that regulate plant’s defense mechanisms. Jasmonic acid (JA) activates defenses against necrotrophs and chewing insects^8^ and Abscisic acid (ABA) and Ethylene modulate plant defenses depending on the type of pathogen attack^9,10^. Hormonal crosstalk fine-tunes plant defense responses against specific attackers. A good example is the crosstalk between SA and JA which is often antagonistic^11^.

The activation of defense pathways as part of induced resistance can be very costly to plants (in terms of resource use)^12^. To optimize resource use efficiency, the regulation of induced resistance needs to ensure that the metabolic burden of the response is less than the potential impact of disease when no defense is mounted. There are substances (e.g. elicitors) that can enhance the efficacy of resistance by assisting a prompt and effective response under the challenge of a pathogen. They are therefore of particular interest as they allow targeted induction of plant resistance during periods of high disease pressure and/or plant susceptibility and thereby increase resource use efficiency. Several studies have shown that elicitor-treated plants show lower infection rates following inoculation with virulent pathogens, but responses can vary greatly between plant species^13^.

In cucurbits, known elicitors include the benzothiadiazole fungicide Acibenzolar-S-methyl, chitin, chitosan, various extracts or compounds of plant origin (e.g. Fucans, Laminarin, Oligogalacturonides), as well as microorganisms (*e.g*. strains of *Bacillus subtilis*)^14^. The extract of the plant Giant Knotweed (RS) was found to be effective against PM fungi such as *Podosphaera xanthii* (syn. *Sphaerotheca fuliginea* (Schlechtend: Fr.) in cucumber^15–17^, *Leveillula taurica* (L.) in tomatoes^18,19^ and *Uncinula necator* (Schw.) Burr. in grapes^20^. PM (caused by *P. xanthii*) is the most common foliar disease of courgettes^21,22^. Various elicitors are now commercially available and marketed as plant strengthening products (e.g. chitin, chitosan, etc.) or plant protection products (e.g. RS), however, our understanding of the molecular and biochemical mechanisms by which elicitors induce plant resistance and how this compares to genetically determined resistance/tolerance is still relatively limited.

The aim of this study was to investigate the effects of RS foliar treatment on courgette genotypes infected with PM. Physiological parameters, disease severity, biochemical and molecular results provide strong evidence, for the first time, that RS elicits plant defenses by inducing the SA pathway.

## Materials and Methods

### Plant material and growth conditions

Two *C. pepo* genotypes (*C. pepo* L. var. *cylindrica*) with different levels of genetic resistance to PM were used; the IR *C. pepo* F1 hybrid, Otto (Syngenta AG, Switzerland) and the S cultivar (cv) Kompokolokitho (Kompo, No. 951, Hellenic Agricultural Organization (HAO)-Demeter, Institute of viticulture and vegetable crops, Pirgos, Greece). Seeds of both genotypes were placed in individual 15 cm pots containing sterile peat substrate for germination, and 1 week later they were transplanted to 2 l pots. When plants reached 3–4 true leaves, they were transplanted into 20 l pots until the end of the experiment(s).

RS (F. Schmidt ex Maxim.) Nakai (Syn. *Polygonum sachalinensis*, *Fallopia sachalinensis*) belongs to the plant family *Polygonaceae*. Currently, the RS extract is not registered in any EU country, although its peer review process has been completed by the European Food Safety Authority (according to Reg (EC) 2009/1107/EC). During RS Review Process by EFSA, five marker components namely resveratrol glucoside: 1.0–8.0% w/w, resveratrol: 0.3–3.0% w/w, emodin glucoside: 2.0–10.0% w/w, emodin: 2.0–8.0% w/w and physcion: 0.5–2.5% w/w were identified to play a key role to its action against PM fungi.

Two formulations of RS were used in this study; a) Milsana, suspension (concentrate composed of 5% ethanolic plant extract), the first commercial RS-product (BIOFA, Munsingen, Germany), and b) Regalia 200SC, a new reformulated RS product commercially available in US (Marrone Bio Innovations), not yet registered in EU. The Regalia 200SC formulation used in courgette was provided by Syngenta AG, Basel, Switzerland. Regalia SC (5% v/v) is applied at 0.5% v/v in 50–100 gallons of water (5 ml^.^l^−1^ formulation of water or 0.25 ml^.^l^−1^ RS, when the dilution is made in 100 gallons of water). Applications of Regalia SC are recommended to commence preventively or when the first disease symptoms are visible.

### Treatments

Plants of both genotypes were grown under a) controlled greenhouse conditions (20–25 °C; Relative Humidity (RH) 70–80%; 16 h light) and b) commercial greenhouse conditions (15–25 °C; RH 55–80%) in two experiments in total. Additionally, plants of the (S) genotype were also grown under controlled and commercial greenhouse conditions.

#### Controlled greenhouse conditions

Foliar sprays of RS were applied to 21 days (d) old courgette plants at the rate of 0.15 ml^.^l^−1^ water. Plants were divided into two treatment groups: (a) extract treated and (b) water treated.

#### Commercial greenhouse conditions

Plants were treated with: 1) RS extract at the rate of 0.15 and 0.3 ml^.^l^−1^ of water, 2) sulphur, formulation Sulfex 80WP (Hellafarm, Athens, Greece) at the rate of 2.5 g^.^l^−1^ of water or 3) myclobutanil, formulation Systhane 20% w/w (Dow AgroSciences, Indianapolis, USA) at the rate of 0.5 ml^.^l^−1^ of water or 0.10 gr a.i.^.^l^−1^ (fungicides were used as reference treatments; a.i. active ingredient). All treatments were applied in a weekly basis until the experiments were ended.

### Experimental design-commercial greenhouse experiments

The experimental designs were as follows:

Commercial greenhouse experiment with both genotypes: A completely randomized, split-plot design with six blocks/replicates was used where (a) genotypes were the main plots (24 plants) and (b) foliar spray treatments were subplots (12 plants).

Commercial greenhouse experiment with S genotype: A single plant plot randomized block design, with six blocks/replicates per treatment was applied (30 plants=5 treatments x 6 plants).

There was 85 cm space between courgette plants within a row and 1 m space between rows in both experiments.

### Inoculation

In the two controlled greenhouse experiments, plants were artificially inoculated with an aqueous conidial suspension of *P. xanthii*, 2 d after treatment applications (freshly collected conidia were suspended in de-ionized water to a concentration of 1.5^.^10^5^ CFU^.^ml^−1^ water).

In the two commercial greenhouse experiments, plants were naturally infected by airborne inoculum of the fungus.

### Assessments

Disease assessments: PM was assessed by visual estimation of the percentage infected leaf area. Disease assessments were performed in the two commercial greenhouse experiments.

Disease severity was assessed weekly on every plant on the upper and lower leaf surface. Percentages were used to calculate the Area Under Disease Progress Curve (AUDPC; %-days) using the equation presented below^23^. The area under the disease progress curve (AUDPC) is a useful quantitative summary of disease intensity over time, for comparison across years, locations, or management regimes and for this reason it was used in this study.

$$AUDPC=\{\mathop{\sum }\limits_{i=1}^{n-1}[({y}_{i}+\,{y}_{i+1})/2]\ast ({t}_{i+1}-{t}_{i})\}$$

#### Where

“*t*” is the time of each assessment, “*y*” is the percentage of affected foliar surface at each assessment and “*i*” is the number of assessments. The variable “t” in this study represents days after transplanting (commercial greenhouse experiment) or days after 1^st^ treatments application (controlled greenhouse experiment).

The AUDPC values were divided by the number of days between the first and the last disease assessment to obtain relative AUDPC values for comparison of PM epidemics between different experiments.

### Crop performance assessments

Crop performance assessments were carried out only in the commercial greenhouse experiment which included both genotypes. The number of leaves per plant was determined at the time of emergence throughout the experiment as a growth indicator.

Leaf chlorophyll measurements: Chlorophyll was measured in the newly emerged leaves (3^rd^ and 4^th^ leaf from the top) and was recorded every two weeks using a SPAD-502 chlorophyll meter device (Konica Minolta Sensing Inc., Japan) which determines the relative amount of chlorophyll present by measuring the absorbance of the leaf in two wavelength regions^24^.

Leaf nitrogen content: Leaf nitrogen content was determined in triplicate at 34 and 62 d after transplant as described by Hornek and Miller^25^.

For the commercial greenhouse experiments: total weight of fruits per plant were recorded during harvest while the plant height (cm) and the number of leaves were recorded weekly.

### Histochemical analyses

For all histochemical analyses, at least 2 leaves from 3 plants of the controlled greenhouse experiment with both genotypes were sampled 12 and 36 h post artificial inoculation (pai) and for each experiment all samples were examined simultaneously.

#### Conidial germination

Microscopic observations were carried out on leaf disks stained with Calcofluor white stain (Fluka, Buchs, Switzerland) before examination.

#### Histochemical detection of hydrogen peroxide

Reactive oxygen species (ROS) production is one of the first events following the recognition of a pathogen by the plant and is induced upon elicitor application^26^. For the *in situ* accumulation of ROS, Hydrogen peroxide (H_2_O_2_) was determined according to the 3, 3’-diaminobenzidine (DAB)-uptake method described previously^27^. Leaf disks were incubated in 0.1% DAB (pH 3.8) overnight in plates covered with aluminum foil at room temperature (25 °C), and then were treated with boiling ethanol at 95 °C to stop the reaction and bleach the disks. Finally, leaf disks were examined under stereoscope for brownish – red precipitates corresponding to H_2_O_2_ accumulation.

#### Callose deposition

Leaf disks were decolorized in 100% ethanol (Merck, Darmstadt, Germany) by boiling at 95 °C for 15 min. Leaf disks were mounted with Lactophenol Blue solution (Fluka, Buchs, Switzerland) and left overnight. Samples were observed after 24 h, under a Leica DM2500 microscope (Leica, Wetzlar, Germany) with blue light excitation filter of 340–380 nm. An AxioCam MRc (Carl Zeiss, Göttingen, Germany), high performance color camera was used for digital documentation. The experiment was repeated twice.

### Confocal laser scanning microscopy

Leaf samples from the callose deposition method (previous paragraph) were mounted between a microscope slide and coverslip in water. Z series were captured using the TCS SP8 confocal laser-scanning microscope (Leica Microsystems CMS GmbH, Heidelberg, Germany). Callose was stained with Lactophenol blue and was excited at 405 nm by using a diode laser. Emission filtering was achieved using a 472–550 nm bandpass filter for Lactophenol blue. Lactophenol blue was excited at a wavelength of 405 nm in the irradiated laser beam. Fluorescence emission of Lactophenol blue was detected in the range of 430–530 nm in wavelength. During image acquisition each line was scanned 5 times and averaged. Image processing, including shadow 3D projection, *in silico* cross section, maximum intensity 3D reconstruction, and surface rendering, was performed using integral functions of the LAS X (Leica) operating software.

### Salicylic acid profiling

Analytical standards of SA ≥ 99.0% and SA-d4 were purchased from Sigma-Aldrich. All the solvents namely acetonitrile, methanol and water were HPLC grade. The stock solution of SA was prepared by accurately weighing 25 mg of each analyte in volumetric flask (certified ‘A’ class) and dissolving in 25 ml methanol. The stock standard solutions were stored at −20 °C. The working standard solution was also stored at −20 °C and before each use was left to reach room temperature. From this working standard solution calibration standards were prepared within the range of 0.001–0.1 mg^.^l^−1^ by serial dilution in methanol 1% HCCOH and addition of SA-d4 at 0.1 mg^.^l^−1^.

Leaf material from the controlled greenhouse experiment with both genotypes was collected 36 h pai. An aliquot of 400 ± 40 mg of frozen sample was weight and 4 ml of extraction solvent (acidified methanol 1% HCOOH) was added. The mixture was shaken for 30 min. in a vertical laboratory shaker (Kottermann) and centrifuged at 4,000 rpm for 5 min (Rotina 360). Before injecting in the chromatographic system, the final solution was filtered through a 0.45 μm disposable cellulose syringe filter. Analysis was carried out using a Varian liquid chromatography system equipped with 2 pumps (Prostar 210) and an automatic sampler (Prostar 420). A Atlantis dc18 Column (100 mm × 2.1 mm × 3 μm, 100 Å) was used at 25 ± 4 °C and the injected volume was 10 μl. The elution gradient was carried out with binary solvent system consisting of MeCN:H_2_O (10:90), 1Mm HCOONH_4_, 0.5% HCOOH (solvent A) and MeCN:H_2_O (90:10), 1MmHCOONH_4_, 0.5% HCOOH (solvent B) at a constant flow rate of 270 μl^.^min^−1^. A linear gradient profile with the following proportions (v/v) of solvent A was applied (t (min)): (0 95), (1 95) (5 40), (10 95) with 5 min for re-equilibration.

LC-MS/MS experiments were performed on a Varian 1200 L triple quadrupole mass spectrometer (Varian). Ionization was performed using an electrospray ionization (ESI) interface operating in the negative mode with the following setting: source temperature 50 °C, drying gas (N2) was heated to 320 °C and pressure 18 psi, nebulizing gas (air) 45 psi. Capillary voltage (CV) and Collision energy (CE) were optimized in infusion experiments using individual standard solutions of SA and SA-d4 at a concentration of 1 μg^.^m^.^l^−1^ diluted in mobile phase A. These solutions were infused at a flow-rate of 10 μl^.^min^−1^ into the mass spectrometer using a Model 11 syringe pump (Harvard Apparatus, Holliston, MA, USA). The following transitions (CV, CE): 137 > 93 (36, 15.5), 137 > 65 (36, 26.5) for SA and 141 > 69 (44, 27.5), 141 > 96 (44, 15.5) for SA-d4. The scanning of the transitions was conducted with a dwell time of 50 msec for each transition. Typical SRM chromatograms for all analytes are presented in Supplementary Fig. 1.

### Phenolic profiling

Leaves were collected form the S plants grown in the second controlled greenhouse experiment at 0, 1, 2 and 4 d pai. The identification and semi-quantification of phenolic compounds was performed by LC-MS using a modified extraction method^28^. One g of each leaf was homogenized with 10 ml of 80% methanol. Samples were purged with N_2_ (3 ml^.^min^−1^) and stored in amber vials protected from light. 10 ml of 6 N HCL was added to each sample. The mixture was transferred to special teflon vessels and placed in a MARS 5 microwave accelerated heating-extraction system (CEM, Carolina, U.S.) at 90 °C for 1 hour for direct hydrolysis of the glycosylated phenolics. The hydrolyzed samples were paper filtered, and the filtrate evaporated. The remaining aqueous phase was partitioned three times with 30 ml ether. The ether fraction was collected in boiling flasks and evaporated at 38 °C until dry. The dry residue was dissolved and collected using 2 ml 20% methanol. The samples were then filtered through HPLC polyester syringe filters and transferred to LC-MS/MS vials for analysis.

For all experiments, the para-coumaric acid (p-hydroxycinnamic acid, 98%), ferulic acid (4-hydroxy-3-methoxycinnamic acid, 99%) and caffeic acid (3, 4-dihydroxycinnamic acid, 99+ %) standards (Acros Organics, Geel, Belgium) were used. Preparation of the stock standard solutions (SSS) was performed by weighting 0.001 g of each standard in a 10 ml volumetric flask and filled to the mark with 20% methanol. A mixed standard solution was prepared every day by transferring 50 μl of each SSS to a 10 ml volumetric flask and filling to the mark with 20% methanol. LC-MS analysis was performed using a Varian 1200 L Series System (Varian, Walnut Creek, USA). Chromatography was carried out on a Varian Polaris 3 μm C18-A (150 mm × 2.0 mm i.d., 5 μm particle size). The mobile phase consisted of water/methanol/formic acid 0.1% v/v. MS/MS analysis was performed using negative electrospray ionization. The mass transitions used for the detection and quantitation of para-coumaric, caffeic and ferulic acids were 163 > 119, 179 > 135 and 193 > 134, respectively. Quantities were estimated with one-point calibration. The exact recorded leaf sample and standard weights were used to express the phenolic compounds in μg^.^g^−1^ fresh leaf tissue. The experiment was repeated twice.

### Quantitative reverse transcription PCR (qRT-PCR)

Plants form both controlled greenhouse experiments, at the developmental stage of 3–4 fully expanded leaves, were sprayed with 0.4 ml^.^l^−1^ of RS or distilled water alone. Artificial inoculation with *P. xanthii* conidia was performed as described above. Leaf material was collected for two different expression analyses. For the first gene expression analysis, leaf material from the controlled greenhouse experiment with both genotypes was collected 36 h pai. For the second gene expression analysis, leaf material from the controlled greenhouse experiment of the S genotype only was collected at four time points: prior artificial inoculation with *P. xanthii* conidia (0 d), and during three consecutive days pai (1, 2 and 4 d). The second and third youngest fully expanded leaves were collected and pooled for each one of four individual plants per treatment, considering each plant as one biological sample. Plant material was frozen in liquid nitrogen and stored at −80 °C until use.

Total RNA was extracted using NucleoZOL (Macherey-Nagel, Duren, Germany) and cDNA was synthesized using 1 μg of total RNA and the PrimeScript™ RT reagent Kit with gDNA Eraser (Takara Bio, Shiga, Japan) according to the manufacturer’s instructions. Real time PCR was performed using a ABI7500 Fast Real-Time PCR system (Applied Biosystems, Waltham, USA) and the KAPA SYBR FAST qPCR kit master mix (KAPA Biosystems, Boston, USA) containing 200 nM of each primer and 1 μl cDNA template (1/20^th^ of cDNA reaction volume). The StepOnePlus thermal cycler real-time system (Applied Biosystems, USA) was used and the following conditions were applied: 10 s at 95 °C, followed by 40 cycles of 95 °C for 5 s and 60 °C for 30 s, with a final 5 s at 95 °C and 30 s at 60 °C. The reactions were run in duplicate and repeated three times. The amplification thermal profile and melt curve profile followed, were as indicated by the manufacturers. Non-reverse-transcribed samples and non-template controls were also included in the relevant PCR runs. The relative gene expression of two experimental runs was determined with the ΔΔ_CT_ method^29^. The information of primers used in this study is provided in Table S1.

### Statistical analysis

Comparisons of phenolics concentrations between RS and control plants for each day were performed using the Independent Samples t Test (SPSS for WINDOWS 16.0).

For the second commercial greenhouse experiment, one-way ANOVA was applied with treatments being the fixed factors.

Effects of foliar treatment (first commercial greenhouse experiment); genotype and foliar treatment on disease severity and plant growth/yield parameters were assessed using ANOVA derived from linear mixed-effects model (lme) of the nlme package^30^ in R^31^. The hierarchical nature of the split-plot design was reflected in the random error structures that were specified as block (replicate)/experiment and block (replicate)/genotype. The normality of the residuals of all models was tested using QQ-plots and found to be normally distributed. Differences and interactions between genotype and foliar treatment were tested using Tukey contrasts in the general linear hypothesis (glht) testing function of the multcomp package^32^ in R. A linear mixed effects model was used for the Tukey contrasts, containing a treatment main effect, with the random error term specified as described above.

## Results

### Differential interactions between courgette genotypes and *P. xanthii*

**Figure 1:**
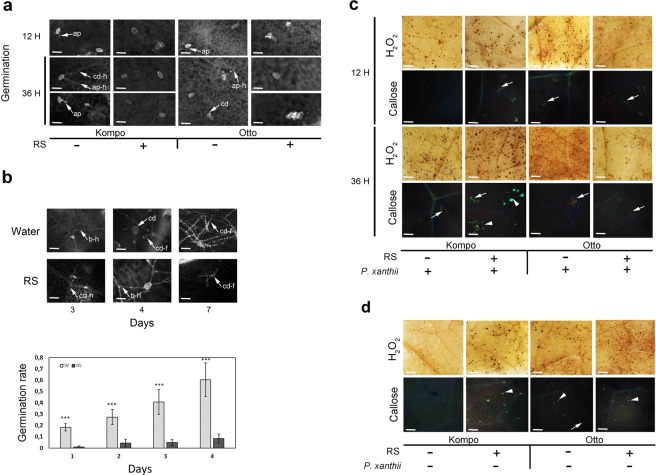
Effect of foliar treatment on proportion of germinated conidia and histochemical analysis after RS application. S (Kompo) and IR (Otto) courgette genotypes were used. (**a**) Conidia germination on leaves of S and IR plants 12 and 36 h pai. The plants were treated with water (− samples) or RS (+ samples). Appressoria (ap) formation was visible, 12 h pai on both S and IR genotypes not treated with RS (−). ap: appressoria; cd: conidia; cd-h: hyphae from conidia; ap-h: hyphae from appressoria. Scale bar: 20 μm. (**b**) Top, conidia germination on leaves of S plants at the indicated time points of non-treated (water) or treated with RS. Bottom, graph indicating the effect of RS application on conidia germination during 4 d pai. 2-way interaction (*P*-value) Day x Foliar Treatment: 0.041. Asterisks indicate significant difference (*P* < 0.001) between RS treated and water treated plants of each day. b-h: branched hyphae; cd: conidia; cd-f: condiophores. Scale bar: 20 μm. (**c**,**d**) Leaves of the S and IR genotypes showing small brownish areas as a result of Hydrogen peroxide production (H_2_O_2_ micrographs) or fluorescence signals detecting callose deposits (callose micrographs) by Lactophenol blue staining and fluorescence microscopy. The plants were treated with water (−), RS (+), water and pathogen inoculation (−/+), RS and pathogen inoculation (+/+). Arrows indicate callose deposites in the periphery of the cells and arrowheads indicate callose granules. Scale bar: 500 μm.

A significant reduction in *P. xanthii* spore germination was observed on RS treated leaves at 12 h pai and was more profound at 36 h pai (Fig. 1a). *P. xanthii* haustoria, were formed on water treated leaves of both genotypes, while lack of appearance was observed on RS-treated leaves of both genotypes. Observations of conidia germination with fluorescent microscopy up to 7 d pai showed that RS application delayed hyphae formation and subsequent conidiophore production when compared with the water treated plants (Fig. 1b). PM developing on leaves of the S Kompo and IR Otto genotypes is shown in Fig. 1a. Appressoria formation was visible 12 h pai on both S and IR genotypes not treated with RS (−), while on RS treated leaves (+) no germinated conidia were observed. 36 h pai secondary hyphae from conidia and appressoria were observed on both S and IR genotypes on RS (−) leaves. No germination was visible on RS (+) treated leaves despite the fact that conidia were viable. Subsequently, percentage germination along with microscopic observations of the germination and host infection process was estimated for a period of up to 7 d pai for the S Kompo genotype (Fig. 1b). Specifically, 3–4 d pai, ca 40% of the conidia had produced branched hyphae on the leaves of not treated with RS (−) plants. Secondary (but less) hyphae had been produced on RS treated leaves (+) but the percentage germination was significantly lower ca 5%. Conidia formation on conidiophores in a colony were observed 7 pai on both RS not treated and treated (−/+) leaves. Despite germination, there was an apparent difference  in the a) number of conidiophores and the density of the mycelium mat observed on controls compared to RS treated leaves and b) percentage of conidia that had germinated to produce hyphae. An overall 80% reduction of germination was observed 4 d pai (Fig. 1b, graph).

The effect of RS application and genetic background (IR hybrid Otto vs S cultivar Kompo) on H_2_O_2_ accumulation in courgette leaves was tested 12 and 36 h pai (Fig. 1c,d). When leaves inoculated with *P. xanthii* were compared, a clear induction of H_2_O_2_ production was detected in RS-treated leaves of the S genotype Kompo 36 h pai compared to non-RS treated leaves (Fig. 1c). Increased H_2_O_2_ production was also detected in RS-treated, non-*P. xanthi* inoculated leaves of the S genotype (Fig. 1d, Kompo +/− sample). In contrast, the IR genotype Otto displayed an enhanced H_2_O_2_ production (compared to the S genotype) in both *P. xanthii* inoculated and non-inoculated leaves and H_2_O_2_ production was not enhanced by RS application compared to non-RS treated (water control) plants (Fig. 1c,d).

Histochemical analysis of callose deposition pattern by fluorescent microscopy identified a similar response pattern to RS-pathogen inoculation for callose deposition. When leaves inoculated with *P. xanthii* were compared, a clear induction of callose formation was detected for the S-genotype Kompo. Non-RS treated leaves (−/+ sample) had almost no callose deposits 12 h pai, when RS-treated plants already showed clear callose deposition (+/+ samples). At 36 h pai, callose deposition in RS-treated plants had progressed to the formation of callose granules (Fig. 1c, Kompo, +/+ sample). Induction of callose formation was also detected in non-*P. xanthii* inoculated leaves after RS treatment (Fig. 1d, +/− sample).

In contrast, when leaves of the IR-genotype Otto were assessed, RS-treatment did not result in a detectable increase in callose formation; similar levels of callose deposition were detected in RS-treated and non-treated leaves from both *P. xanthii* inoculated and non-inoculated plants 12 h pai (Fig. 1c,d). However, at 36 h pai, when conidia had germinated on both RS-treated (+/+ sample) and non-treated RS (−/+ sample) leaves, lower levels of callose deposition than at 12 h pai were observed (Fig. 1c). Callose examination of IR non-inoculated plants had a similar pattern to ROS generation. Both water (−/− sample) and RS (+/− sample) treated leaves showed a similar enhanced callose production (Fig. 1d).

**Figure 2:**
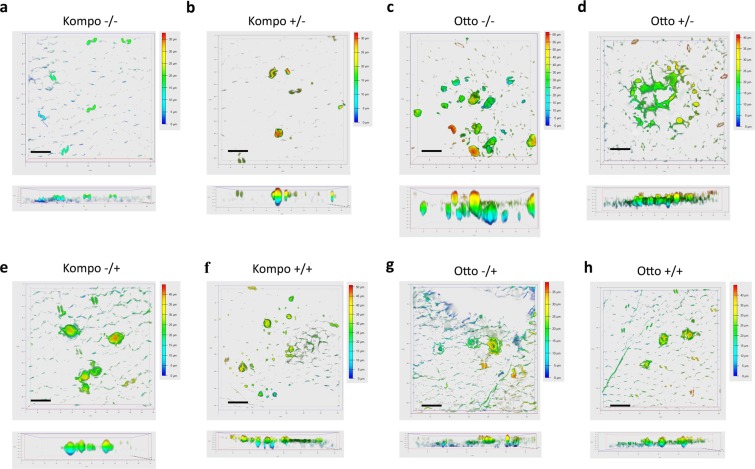
Callose formation varies between genotypes and RS treatment. **(a–h)** Confocal z-stacks of Lactophenol blue stained leaves of the same genetic material and treatments as in Fig. 1. Vertical section is presented under each z-stack depicting callose granule length. Colored scale bar: depth coding scale bar of the z-stack. Scale bar: 100 μm.

In order to examine the micromorphology of callose deposits, leaf segments from all treatment combinations were examined with confocal microscopy at 36 h pai. Callose was shown to deposit in epidermal cell periphery or form granules which represented accumulated callose deposition that had penetrated epidermal cells (Fig. 2). Callose granule formation was more profound in the Otto control samples where granules could reach 65 μm in length (Otto, −/−, Fig. 2c). This deposition could only be compared with Kompo, +/+ samples, where granules had a length of up to 48 μm (Fig. 2f).

**Figure 3:**
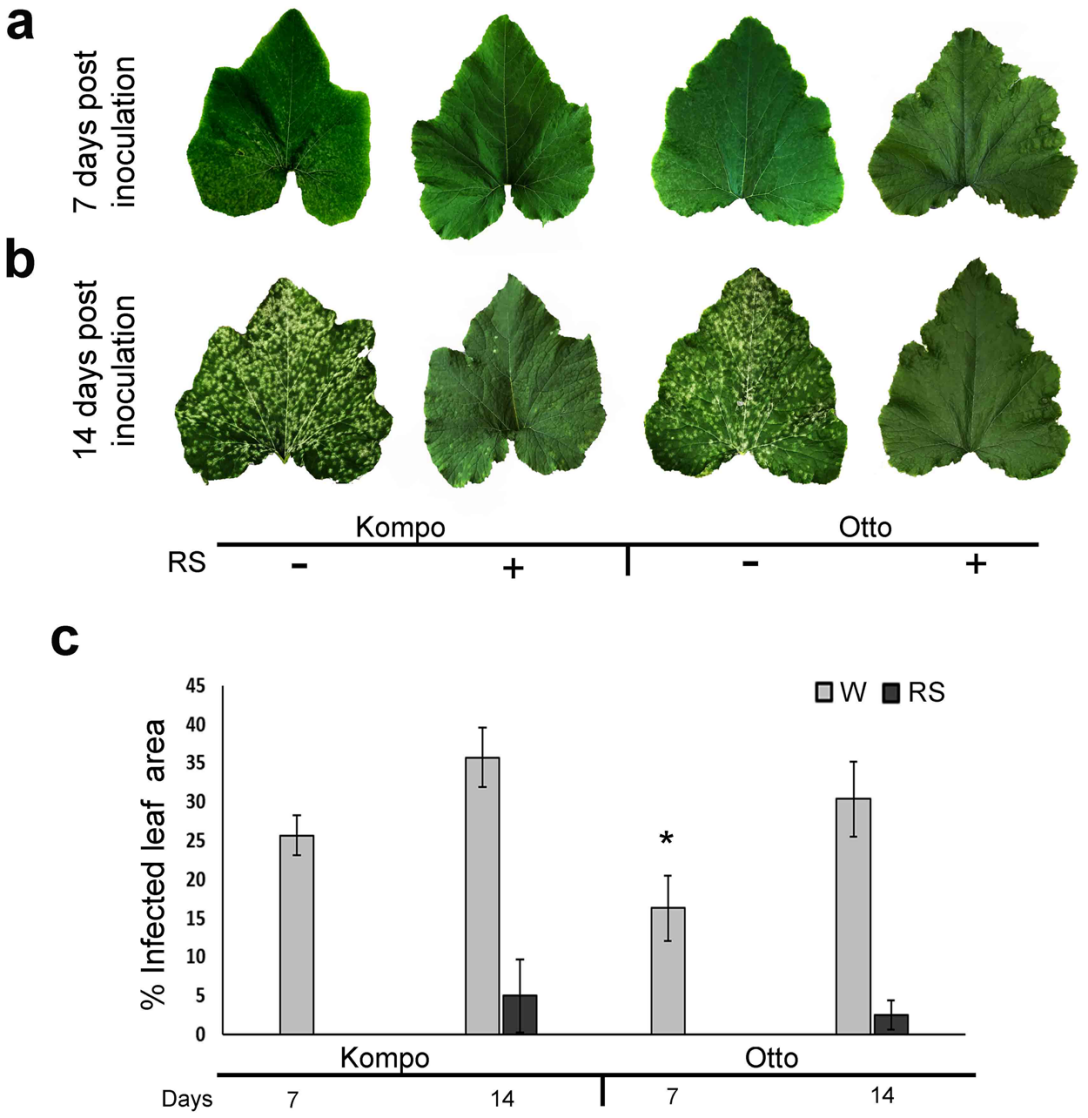
PM disease symptoms on leaves of S (Kompo) and IR (Otto) courgette genotypes. (**a)** Indicated pattern of whitish powdery colonies of *P. xanthii* spread over a leaf of the S genotype 8 d pai (Kompo −/+), while the RS treated leaves (Kompo, +/+; Otto +/+) and the IR leaf (Otto −/+) show none or reduced colony formation, respectively. (**b)** The same pattern of infection is maintained at 12 d pai. All leaves in a, b are at the same developmental stage. (**c)** PM severity (mean % leaf area damaged ± 1 SD) on leaves of the IR and S genotypes treated with water (−) or RS (+) at the indicated time points. For each sample six plants were used and from each plant the average of % infected area of all leaves was calculated. Error bar represents standard deviation of mean. Statistical significance was based on Student’s t test. *P ≤ 0.05.

Visual assessment of PM development on courgette leaves 7 and 14 d pai showed significant differences of disease symptom development between (a) courgette genotypes and (b) RS-treated and untreated plants. 7 d after *P. xanthii* inoculation, fungal colonies were visible as small white powdery spots on the leaf surfaces of controls (water treated) plants of the S genotype Kompo, while colonies on the leaf surfaces of control plants of the IR genotype Otto were less distinct, bearing less conidiophores and mycelium (Fig. 3a). In contrast, no *P. xanthii* colonies could be detected on both S and IR genotype plants treated with RS 14 d pai (Fig. 3b). PM severity on RS-treated plants of both genotypes grown at the same time and under the same conditions to those sampled for analysis, was significantly reduced for up to 14 d pai when compared to water treated plants (Fig. 3c).

### *In planta* efficacy of RS extract against *P. xanthii*

Two greenhouse experiments using commercial courgette agronomic protocols were performed.

In the first greenhouse experiment, RS foliar treatments were compared to both a positive (foliar application of a sulphur fungicide) and negative (foliar application of water) control treatments using both the S (Kompo) and IR (Otto) courgette genotypes. The results showed that RS foliar treatment significantly reduced PM severity (AUDPC values were used for the calculation, Table 1) on the upper leaf surface by 54.4 and 72% in plants treated with 0.15 and 0.3 ml^.^l^−1^ RS, respectively. There was no statistically significant difference in PM severity between 0.3 ml^.^l^−1^ RS and Sulphur treated plants (Table 1). There was no significant main effect of genotype and no significant interaction between genotype and foliar treatment on PM severity, but it should be noted that numerically PM-severity was ~20% lower on the lower leaf surface of the IR genotype (Table 1). Also, overall the IR genotype produced a significantly higher fruit yield (140%) compared to the S genotype (Table 1).

**Table 1:** Effect of, and interactions between, genotype and foliar treatment on powdery mildew severity (Area Under Disease Progress Curve; AUDPC) on the upper, lower leaf surface and fruit yields of the S and IR genotypes treated with RS, fungicide or water (2-factor and 1-factor ANOVA).

Means ± SE	AUDPC(%-days) Upper Leaf Surface	AUDPC (%-days) Lower Leaf Surface	Yield Fruit g plant^−1^	AUDPC(%-days) Upper Leaf Surface	AUDPC (%-days) Lower Leaf Surface	Yield Fruit g plant^−1^
*Greenhouse Experiment 1*				*Greenhouse Experiment 2*		
**Genotype (n = 24)**						
S	15.4 ± 2.27	8.8 ± 0.91	66 ± 13.76	—	—	—
IR	15.9 ± 2.24	6.9 ± 1.13	140.6 ± 14.03	—	—	
**Foliar treatment (n = 12)**						
Control (Water)	31.8 ± 1.86 a	5.7 ± 0.93 b	69.5 ± 28.84	35.7 ± 3.08 a	26.3 ± 2.68 a	109.2 ± 29.62
RS (0.3 ml l^−1^)	8.9 ± 1.09 c	5.2 ± 0.42 b	112.7 ± 9.75	17.4 ± 1.34 b	19.4 ± 1.35 b	245 ± 56.49
RS (0.15 ml l^−1^)	14.5 ± 1.59 b	9.1 ± 1.6 a	122.9 ± 24.62	18.6 ± 2.45 b	19.5 ± 2.25 b	158.3 ± 48.07
Sulphur (2 g l^−1^)/myclobutanil (0.10 gr a.i. l^−1^)	7.4 ± 1.15 c	11.4 ± 1.8 a	108.1 ± 20.92	15.6 ± 1.17 b	17.3 ± 1.44 b	248.3 ± 62.76
**ANOVA**						
**Main effects**						
Genotype	ns	ns	**	NR	NR	NR
Foliar treatment	***	**	ns	***	**	ns
**Interactions**						
Genotype x Foliar Treatment	ns	ns	ns	NR	NR	NR

Within each column, means followed by the same letter are not statistically different (General Linear Hypothesis Test (GLHT); P < 0.05); ns, not significant; **P < 0.01; ***P < 0.001.

NR, not relevant.

In the second greenhouse experiment only the S genotype was used and results showed similar trends to the first experiment, with both RS application rates resulting in a significant (50%) reduction of disease severity on the upper leaf surface, compared to water treated control plants, similar to the reduction achieved by the synthetic chemical fungicide myclobutanil at the label recommended rate (Table 1).

**Figure 4:**
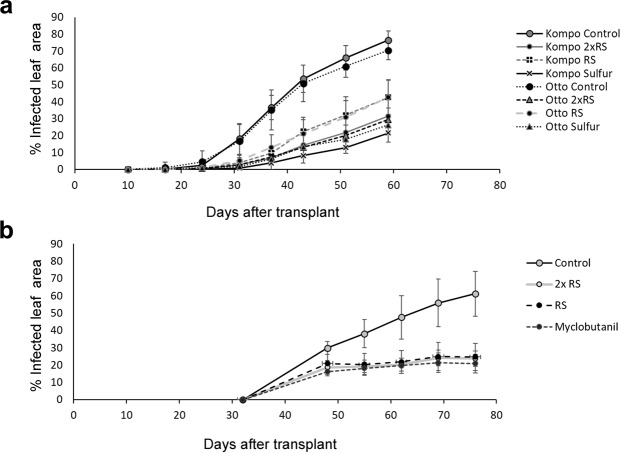
RS foliar application reduces significantly PM severity. (**a)** PM severity (mean % leaf area damaged ± 1 SD) on leaves of the IR (Otto) and S (Kompo) genotypes treated with water (−/+) or two different doses of RS (+/+) and with a fungicide (Sulphur) at the indicated time points. (**b)** PM severity on leaves of the S genotype treated with water (−/+), 2 different doses of RS (1×, 2×) and with a fungicide (Myclobutanil) at the indicated time points. Error bar represents standard deviation of mean.

When data for the S genotype from both experiments were analyzed together by 2-factor ANOVA only a significant effect of treatment was detected, while there was no significant main effect of experiment (and associated differences in greenhouse conditions) and no interaction between experiment and treatments (Table 2). On average (over the 2 experiments) RS at the higher application rate reduced PM severity by 52% and increased yields by 90% in the S cultivar (Table 2). When the disease progress curves of plants form the S and IR genotype receiving the same foliar treatment were compared, no significant differences were detected (Fig. 4).

**Table 2:** Effect of, and interactions between, experiment and foliar treatment on powdery mildew severity (Area Under Disease Progress Curve; AUDPC) on the upper leaf surface and fruit yields of courgette plants (2-factor ANOVA; S genotype).

Means ± SE	Fruit Yield g plant^−1^	AUDPC-Upper Leaf Surface (%-days)
**Experiment** (n = 48)		
Experiment 1	170.8 ± 28.48	23.9 ± 2.41
Experiment 2	101.7 ± 13.26	18.4 ± 1.86
**Foliar treatment** (n = 18)		
RS (0.3 ml l^−1^)	156.8 ± 24.14 a	11.7 ± 1.27 c
RS (0.15 ml l^−1^)	134.7 ± 22.46 ab	15.9 ± 1.38 b
Control (Water)	82.7 ± 21.57 b	33.1 ± 1.62 a
**ANOVA**		
**Main effects**		
Experiment	ns	ns
Foliar treatment	*	***
**Interactions**		
EX x FO	ns	ns

Within each column, means followed by the same letter are not statistically different (GLHT test; P < 0.05): ns, not significant; *P < 0.05; ***P < 0.001.

Significant main effects of both courgette genotype and foliar treatments on leaf nitrogen (34 d after transplanting) and chlorophyll content (24 d after transplanting) were also detected and there were significant interactions between genotype and foliar treatment for N-content (62 d after transplanting) and for leaf chlorophyll (24 d after transplanting) (Table 3). The S genotype had a significantly lower N-content 34 d after transplanting and both use of the IR genotype and the higher rate RS application resulted in significantly higher chlorophyll contents 24 d after transplanting (Table 3). Also, there was a significant main effect of genotype on both growth parameters assessed in this study, with the S-genotype producing significant higher numbers of leaves and taller plants (Table 3).

**Table 3:** Effect of, and interactions between, genotype and foliar treatment (RS, Sulphur) on nitrogen content (%), leaf chlorophyll content (SPAD values), number (No) of leaves and plants height (means ± SE) of squash plants (2-factor ANOVA).

Factor		N-content (%)		Leaf Chlorophyll (SPAD values)					
		34 d after transplant	62 d after transplant	24 d after transplant	38 d after transplant	53 d after transplant	82 d after transplant	Total No of Leaves	Plant Height (cm)
**Genotype**									
(n=48)	IR	5.20 ± 0.11	3.77 ± 0.12	36.9 ± 0.4	39.09 ± 0.50	36.89 ± 0.64	28.49 ± 0.70	20.27 ± 0.17	37.63 ± 0.92
	S	5.49 ± 0.09	3.85 ± 0.13	34.1 ± 0.4	38.90 ± 0.48	36.60 ± 0.63	28.52 ± 0.95	21.35 ± 0.23	45.93 ± 0.91
**Foliar Treatment**									
(n=24)	1. RS (0.3 ml l^−1^)	5.26 ± 0.16	3.73 ± 0.18	36.95 ± 0.53 a	40.04 ± 0.76	37.23 ± 0.93	31.48 ± 1.36 a	20.71 ± 0.27	41.35 ± 1.17
	2. RS (0.15 ml l^−1^)	5.40 ± 0.13	3.63 ± 0.17	35.88 ± 0.53 ab	37.92 ± 0.60	37.80 ± 0.94	28.03 ± 1.34 b	20.25 ± 0.31	42.29 ± 1.41
	3. Control (water)	5.39 ± 0.15	3.95 ± 0.13	34.42 ± 0.69 b	39.55 ± 0.71	35.01 ± 0.89	26.59 ± 0.97 b	21.21 ± 0.32	41.56 ± 1.73
	4. Sulphur (2 g l^−1^)	5.33 ± 0.14	3.91 ± 0.21	34.68 ± 0.64 b	38.49 ± 0.64	36.95 ± 0.74	27.86 ± 0.72 b	21.08 ± 0.29	41.90 ± 1.86
**ANOVA results**									
**Main effects**									
Genotype		*	NS	***	NS	NS	NS	***	***
Foliar Treatment		NS	NS	**	NS	T	*	T	NS
**Interactions**									
Genotype x Foliar Treatment		NS	*	*	NS	NS	T	NS	NS

Within each column, means followed by the same letter are not significantly different (GLHT test; *P* < 0.05); NS, not significant; T *P* < 0.1; *P < 0.05; **P < 0.01; ***P < 0.001.

### Effect of RS extract on marker genes related to biotic stress

In order to understand the effect of RS on plants and PM severity observed in glasshouse trials, the expression levels of major genes related to biotrophic fungi infection were analyzed. The genes tested were the SA receptor *NPR1*, the pathogenesis-related genes *PR1* and *PR2* and a major regulator of the phenylopropanoid pathway, *PHENYLALANINE AMMONIA-LYASE* (*PAL*) gene.

**Figure 5:**
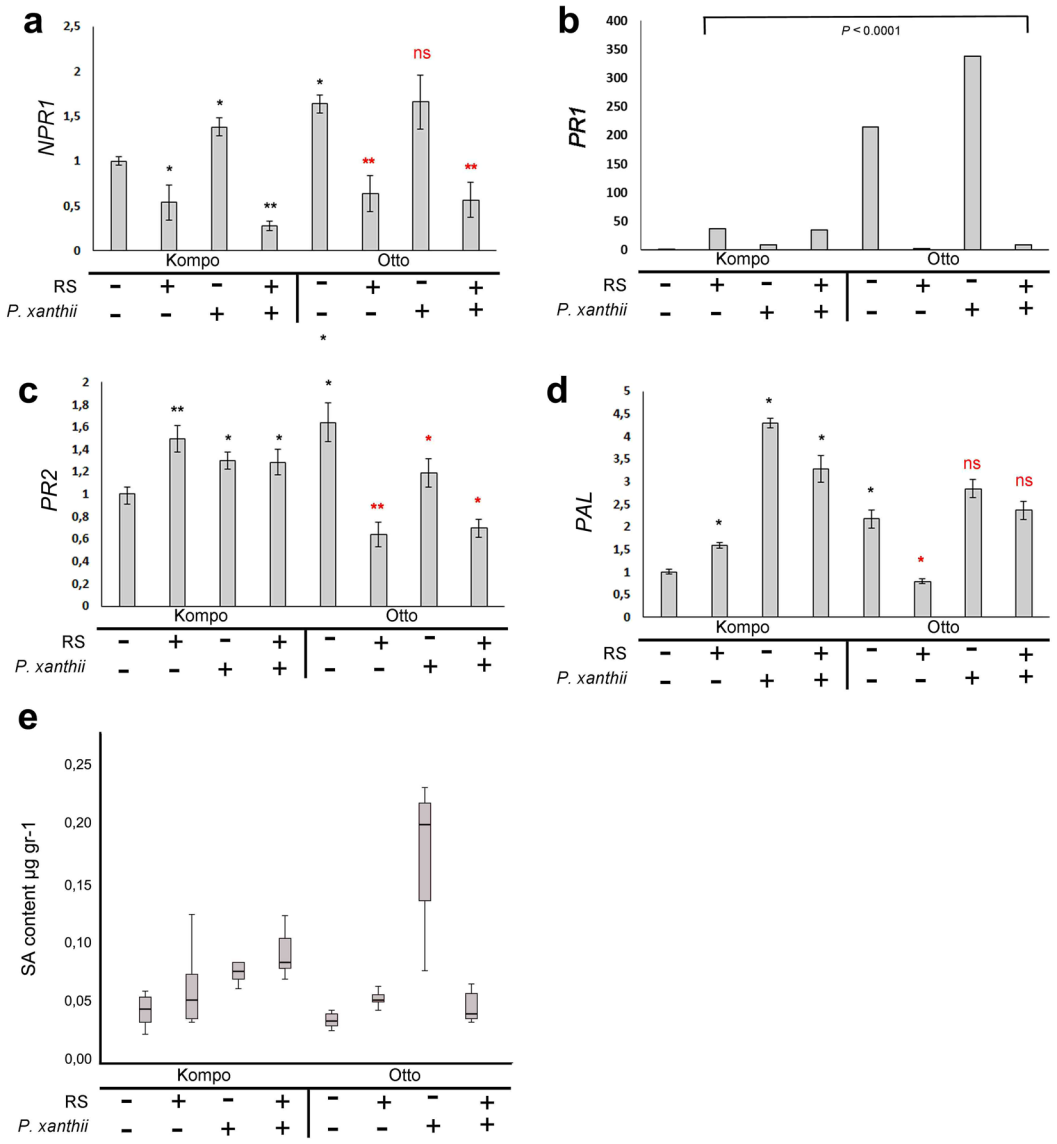
RS treatment positively modulates SA-responsive gene expression and SA content. Expression of (**a**) *NPR1*, (**b**) *PR1*, (**c**) *PR2* and (**d**) *PAL* in leaves of S (Kompo) and IR (Otto) courgette genotypes 36 h pai. The plants were treated with water (−/−), RS (+/−), water and pathogen inoculation (−/+), RS and pathogen inoculation (+/+). The expression levels of all genes were determined relative to *EF1a* on three biological replicates. Error bar represents standard deviation of mean. Statistical significance was based on Student’s t test. *P ≤ 0.05; **P ≤ 0.01; ns: non-significant. Black color of significance markers indicates correlation with Kompo (−/−) sample and red color indicates correlation with Otto (−/−) sample. (**e**) Leaf samples as from the above expression analysis were tested by HPLC-MS-MS for SA content. The experiment was repeated three times with similar results.

Gene expression analyses were performed in both genotypes 36 h pai to investigate whether the observed histochemical, phenotypic and phenological disease severity assessments were correlated with altered gene expression. Transcript accumulation of the SA receptor *NPR1* indicated downregulation in *P. xanthii* inoculated plants treated with RS in both genotypes when compared with inoculated water treated ones (Fig. 5a). Examination of *PR2* gene expression revealed a similar pattern with *NPR1* in the IR genotype, while a modest but significant increase in transcript levels was detected in all S genotype samples when compared with the water treated S ones (Fig. 5c). An interesting pattern was observed when transcript levels of the *PR1* gene were assessed. In the S genotype, RS treatment induced significantly *PR1* transcription (25-fold) in pathogen inoculated plants, while in the IR genotype, RS application caused significant downregulation of *PR1* gene expression (from 330 to 10-fold) in the inoculated plants. A major distinguishable difference between the two genotypes was *PR1* expression levels in negative control (water treated) plants. Comparison of IR (Otto, −/− sample) to S plants (Kompo, −/− sample) showed that there is a highly enhanced *PR1* expression (214-fold) in IR genotype when plants were not inoculated with a pathogen (Fig. 5b).

*PAL* gene transcript levels were downregulated in RS treated IR plants, while they remained unaltered in the other treatment combinations (Fig. 5d). When the S genotype was tested, *PAL* transcript levels showed significant (50%) upregulation after RS application (Kompo, +/− sample). Upregulation of transcript levels was enhanced 3.2-fold after pathogen inoculation (Kompo, +/+ sample), even though it was 50% lower compared to water treated inoculated plants (Kompo, −/+ sample) (Fig. 5d). Examination of SA levels in all samples revealed that RS application on the S genotype induced SA accumulation prior and after pathogen infection, while RS application had no significant effect in the IR genotype. Interestingly, the SA concentration in water treated IR plants was relative lower than those found in water-treated S plants but substantially increased after pathogen infection (Otto −/+ sample) (Fig. 5e).

**Figure 6:**
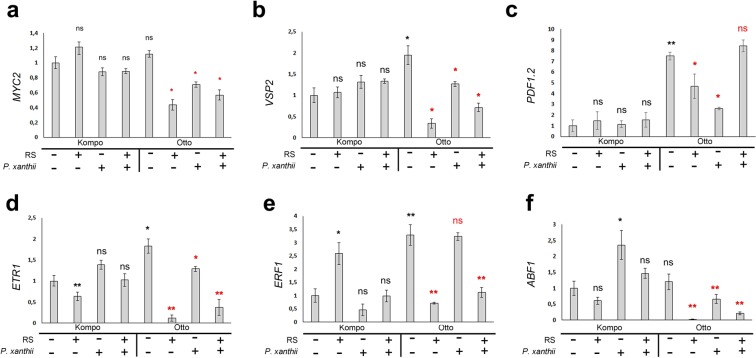
Major JA, Ethylene and ABA pathway genes are not correlated with RS application. Expression of (**a**) *MYC2*, (**b**) *VSP2*, (**c**) *PDF1.2*, (**d**) *ETR1*, (**e**) *ERF1* and (**f**) *ABF1* in leaves of S (Kompo) and IR (Otto) courgette genotypes 36 h pai. The plants were treated with water (−/−), RS (+/−), water and pathogen inoculation (−/+), RS and pathogen inoculation (+/+). The expression levels of all genes were determined relative to *EF1a* on three biological replicates. Error bar represents standard deviation of mean. Statistical significance was based on Student’s t test. *P ≤ 0.05; **P ≤ 0.01; ns: non-significant. Black color of significance markers indicates correlation with Kompo (−/−) sample and red color indicates correlation with Otto (−/−) sample.

To get a more thorough understanding of the plant’s response to RS application, major genes corresponding to other hormonal signaling pathways were tested. Firstly, *JASMONATE INSENSITIVE 1* (*MYC2*), a master regulator of the JA-signaling pathway and a principal component of JA crosstalk with other hormonal pathways was tested (Fig. 6a). *MYC2* showed no significant changes in the expression profile in the S genotype. A limited downregulation of expression was observed in all infected and/or RS treated samples in the IR genotype, however, the difference was not significant. The same pattern was observed when the ABA and JA responsive gene *VEGETATIVE STORAGE PROTEIN 2* (*VSP2*) and the JA and ethylene responsive gene *PLANT DEFENSIN 1.2* (*PDF1.2*) were tested (Fig. 6b,c respectively). However, a substantial difference in *PDF1.2* gene expression was observed between non-inoculated, untreated (water) control plants of the S (Kompo, −/− samples) and IR (Otto, −/− samples) genotypes. The IR showed a 7-fold increased expression compared to S (Fig. 6d). The *ETHYLENE RESPONSE 1* (*ETR1*), *ETHYLENE RESPONSE FACTOR 1* (*ERF1*) and the *ABSCISIC ACID RESPONSIVE ELEMENT-BINDING FACTOR 1* (*ABF1*) genes were also tested. *ETR1* and *ERF1* are known to participate in ethylene signaling and to be induced as part of plant’s defense against pathogens^33^, while *ABF1* is an abiotic stress marker gene, also known to mediate biotic stress responses^34^. RS application was shown to downregulate the expression pattern of *ETR1* in both genotypes, with and without pathogen inoculation (Fig. 6e). Even though RS application alone induces *ETR1* and *ERF1* gene expression in uninfected S samples (Kompo, −/+ samples), in Kompo, +/+ samples, pathogen inoculation does not affect gene expression in S samples and downregulates the expression of both genes in IR samples (Fig. 6e). Interestingly, in control IR plants (Otto, −/− samples) *ERF1* gene expression was 2.5-fold higher than in control S plants (Kompo, −/− samples). *ABF1* was induced by pathogen infection and its transcript levels followed a similar pattern as described for the other two genes (Fig. 6f).

**Figure 7:**
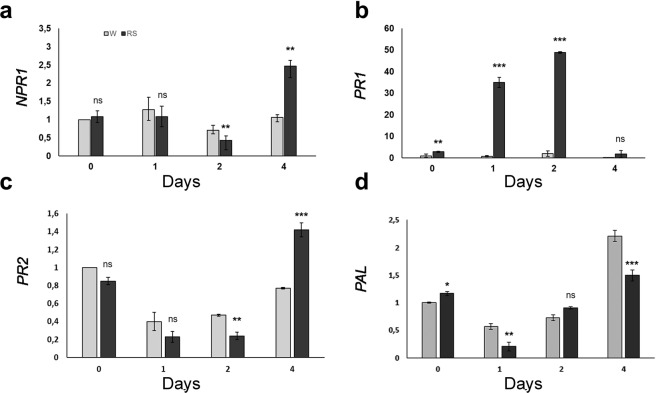
RS application positively regulates *NPR1*, *PR1* and *PR2* expression through time after *P. xanthii* inoculation in S (Kompo) plants. Expression of (**a**) *NPR1*, (**b**) *PR1*, (**c**) *PR2* and (**d**) *PAL* in inoculated leaves of S genotype at the indicated four time-points. The expression levels of all genes were determined relative to *EF1a* on three biological replicates. The plants were treated with water (W) or RS extract (RS) and inoculated with *P. xanthii* conidia at 0 d. Error bar represents standard deviation of mean. Statistical significance was based on Student’s t test. **P ≤ 0.01; ***P ≤ 0.001; ns: non-significant.

The effect of RS application on genes of the SA pathway was also studied at different times after *P. xanthii* inoculation. The expression analysis of the *NPR1*, *PR1*, *PR2* and *PAL* genes was performed at 0, 1, 2, and 4 d pai on S plants. Our results showed that just before plant inoculation with *P. xanthii* conidia at 0 d, almost all tested genes showed a basal/constitutive level of expression in both RS and water treated plants, with levels of expression being between 0.8 to 1.2-times higher in RS-treated compared to water treated control plants (Fig. 7a,c,d). Following inoculation with *P. xanthii* conidia, different patterns of gene expression were detected. *NPR1* and *PR2* gene expression was reduced during the first 2 d pai in RS and water treated plants. However, 4 d pai, an upregulation of both gene transcripts (2.5 and 1.4-fold for *NPR1* and *PR2*, respectively) was observed in RS treated plants compared to water treated control plants (Fig. 7a,c).

The pattern of expression observed for the *PAL* gene was as follows. Reduced gene expression was observed during the first 2 d pai in both RS treated and water treated plants (Fig. 7d). At 4 d, both samples showed an upregulation in gene expression; the relative increase in *PAL* expression in RS treated was lower than in water treated control plants (1.5-fold and 2.8-fold and in RS and water treated plants, respectively).

Examination of *PR1* gene expression showed upregulation in RS treated plants prior and after pathogen inoculation (Fig. 7b). The 2.9-fold enhanced transcript accumulation in RS treated plants at 0 d was increased to 35-fold, 24 h pai and 49-fold 2 d pai. *PR1* transcript accumulation then declined to levels similar to those observed in negative (water treated) control plants at 4 d pai (Fig. 7b).

### Concentrations of phenolic compounds in courgette leaves treated with RS

Literature reports show that RS application induces the accumulation of secondary metabolites in *P. xanthii* inoculated cucumber plants^15^. These secondary metabolites act as phytoalexins and assist in defense responses to pathogen attack.

**Figure 8:**
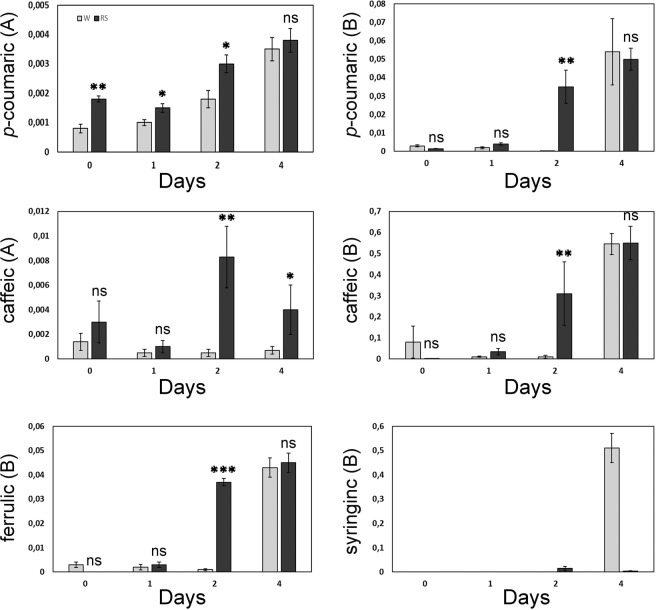
RS application induces the production of phytoalexins prior and after pathogen inoculation in S (Kompo) plants. Leaf samples were tested by HPLC-MS-MS for phytoalexin content. Mean concentrations (μg x g^−1^ fresh leaf tissue) of *p*-coumaric and caffeic acids (±SE) in their free and aglycone forms and ferulic and syringic acids in their aglycone forms in leaf tissues sprayed with *R. sachalinensis* extract (0.4 ml l^−1^) and water before (0), 1, 2 and 4 d after artificial inoculation with *P. xanthii*. (**A**) Represents results for free phenolics and (**B**) for aglycone phenolics obtained after hydrolysis (Student’s t-test: n.s., not significant; *P < 0.05; **P < 0.01; ***P < 0.001). The experiment was repeated three times with similar results.

The effect of RS treatment on the concentration of major phenolic compounds was therefore assessed in leaves of the S genotype (Kompokolokitho), which had shown a reduction in mildew severity in response to RS treatment following *P. xanthii* conidial inoculation. There was rapid increase in the concentrations of several free (A) and aglycone forms (B) of phenolic compounds in RS-treated compared to water-treated negative control plants 2 d pai, with concentrations of p-coumaric and caffeic acids in free and aglycone forms (A and B) found to be 1.7, 71 and 11.8, 23.2 times higher in RS treated compared to water treated plants (Fig. 8). However, 4 d pai, concentrations of *p*-coumaric acid (A and B), caffeic acid (B) and ferrulic acid were at similar levels in RS-treated and water treated plants. Also, concentrations of free forms of caffeic acid (A) were significantly higher in RS-treated plants, while syringic acid aglycone forms (B) concentrations were higher in control plants (Fig. 8). It should be noted that concentrations of free phenolics (A), p-coumaric and caffeic acids, were present in traces only.

## Discussion

Genetic resistance to PM is not known to occur naturally within *Cucurbita* species and is only rarely occurring in *Cucurbita moschata* varieties. Approaches -other than pesticides applications- to control powdery mildew include the use of IR genotypes (deriving from introgression of a major resistance locus from the wild species *Cucurbita okeechobeensis* subsp. Martinezii)^35^ and the elicitation of the plant’s intrinsic defense mechanisms. We studied the effect of RS application on PM progress in S and IR courgette genotypes and described its mode of action. Using an array of physiological, cellular and molecular approaches we showed that RS can have a lasting protective effect against PM in courgettes by enhancing plant defense responses at different levels.

The study of the genotype effect on disease progress showed that the IR genotype did not result in a significant reduction of disease severity on leaves. However, the IR plants produced substantially higher yield compared to the S genotype, suggesting that it would be important to quantify the potential contribution of PM-tolerance on yield in future trials. In addition, IR leaves bared less abundant mycelium compared to the S genotype, a finding which can be attributed to less *P. xanthii* conidia production on cucumber genotypes with a form of genetic resistance^36^.

Previous studies have shown that application of RS extract on cucumber leaves can significantly reduce PM severity^16,17,37^ which was attributed to induction of plant defense mechanisms. Induced resistance has been associated with increased enzymatic activity (of chalcone synthase and isomerase, peroxidases, β−1,3-glucanases)^38^, accumulation of phenolic compounds^15,28,39^, collapse of fungal structures and deposition of dense materials around fungal haustoria^40,41^. Enhanced callose deposition and ROS accumulation in the IR genotype on one hand and the RS treated S genotype on the other hand indicate that reduction of conidial germination could be attributed to “local induction of resistance”; findings that are in agreement with previous studies in cucumbers^40^. Accumulation of reactive oxygen species may induce plant resistance by triggering a hypersensitive reaction in plant cells and/or serving as signaling molecules for defense responses, as was shown in RS treated wheat plants^42^. Furthermore, research with *Arabidopsis thaliana* lines overexpressing a callose synthase gene, demonstrated that increased production of callose resulted in enhanced penetration resistance and reduced haustoria formation^43^, corroborating our results. So far, inhibition of conidial germination by RS has not be linked to fungitoxic properties of the extract^38^. Although some of RS key components, such as resveratrol, emodin and physcion, have been shown to have antimicrobial action against pathogenic fungi causing powdery and downy mildews^44–46^, all the aforementioned studies with RS however, concluded that the reduction of PM incidence, was mainly attributed to a modulation of the plant’s defense responses.

We further studied the molecular responses of courgette plants to RS application, prior to and after artificial inoculation of plants. SA is a plant hormone that plays pivotal role in plant defense against biotrophic and semi-biotrophic pathogens. Treatment of S plants with RS extract challenged *NPR1* expression with a simultaneous *PR1* upregulation at the first 2 d of pathogen infection following a reverse pattern at 4 d. SA content also increased in RS treated plants with and without pathogen inoculation. Our results are consistent with the activation cycle and feedback of *PR1* by NPR1, where direct SA binding to NPR1 protein in the cytoplasm translocates NPR1 to the nucleus for *PR1* activation^47,48^. Consequently, we can suggest that SA production by RS application rapidly activates NPR1 protein translocation to the nucleus for *PR1* activation, leading to decrease of *NPR1* transcripts and simultaneous increase of *PR1* transcript levels, until the next cycle of activation takes place. Moreover, studies on *Arabidopsis* mutants, such as *salicylic acid induction deficient2* (*sid2*), *enhanced disease susceptibility5* (*eds5*), which are deficient in pathogen-induced SA accumulation, and on *npr1* mutant, exhibit strongly reduced *PR1* expression and increased susceptibility to biotrophic pathogens findings that are also consistent with our findings^49–51^.

Additionally, upregulation of the SA responsive *PR2* and *PAL* genes, reinforce the perception of RS-induced activation of the SA defense mechanism. Furthermore, *PAL* gene expression positively correlated with increased SA content and free *p*-coumaric acid levels, indicating elicitation of resistance in RS treated plants. *PAL* is involved in the biosynthesis of SA through the phenylalanine ammonia-lyase (PAL) pathway which also involves the biosynthesis of *p*-coumaric in the stage of conversion of phenylalanine to ammonia and cinnamate^52,53^. The induced defense effect was also apparent in phytoalexin production and was demonstrated by increased production of major defensive secondary metabolites 2 to 4 d pai in RS treated plants. Our results are in agreement with previous studies showing that RS application increased the activities of PR and PAL proteins^54,55^ resulting in enhanced resistance of cucumber plants to PM.

Following microbe attack, plants synthesize a complex blend of SA, JA, and ET hormones, with the specific combination being case specific. These hormonal signals crosstalk in order to modulate effective defense mechanisms against pathogen attack^56^, with SA having a pivotal role in defense against biotrophic pathogens and JA/ET being critical to defense against necrotrophs^57^, suggesting a binary model with SA and JA/ET having antagonistic and opposite roles. Besides these classical plant defense hormones, ABA and other hormones including brassinosteroids, gibberellins and auxins, have emerged as additional players in plant-microbe interactions^56^. ABA appears critical for both abiotic and biotic stress defense mechanisms^34^ and possibly a central general modulator of the regulatory crosstalk^58^. Our evaluation of RS inductive capacity to other hormonal pathways revealed SA-specific dynamics of the RS extract since we did not observe any upregulation in expression of the tested genes corresponding to the other hormonal pathways.

On the other hand, the combined effect of the genotype and the RS extract application led to a dramatic reduction of plant’s defense responses in RS-treated IR plants. We speculate that RS treatment “over-stimulated” defense responses in IR plants, triggering post-translational modifications that had an opposite effect on plant’s natural resistance. Nevertheless, the enhanced expression profile of the IR control plants (water treated) compared to the S ones, suggests that resistance could be attributed to an overall genetic reinforcement of plant’s responses. This could explain the low SA content compared to increased *PR1* gene expression, since *PR1* is shown to be activated and by other hormonal pathways except SA^59^.

Exogenous SA application can induce ROS production, PR gene expression and immunity against various pathogens with biotrophic or hemi-biotrophic lifestyles^60^. Our findings provide strong evidence that the RS extract stimulates plant defense and acts *via* the SA pathway setting plants in a priming state for imminent pathogen attack. The gene expression analysis correlates with the cytological stains of callose deposits and ROS production, which show stimulation of defense responses. There are several synthetic products that act as functional SA analogs, activating the SA-dependent defense pathway in many plant species^61,62^, however, only naturally seaweed extracts have been reported to have this capacity against fungal pathogens of strawberry^63^. Here, for the first time, this property is being attributed to the natural plant extracts from giant knotweed, formulated today as Regalia^®^ Bioprotectant concentrate.

The results presented in this study together with similar research approaches in the field of plant elicitors, could aid the development of a new generation of plant protection products of plant origin, since they elucidate their mode of action(s) and effectiveness, which is required for both the safety evaluation and registration process. Furthermore, they provide farmers and growers with accurate information about the benefits and limitations of plant extract-based products/strategies. For example, results from this study suggest that integration of RS treatments with the use of IR varieties/hybrids may not be cost effective in terms of mildew control in commercial production.

## Main Conclusion

Physiological and gene expression analysis in courgette plants sprayed with *Reynoutria sachalinensis* extract revealed that it induces SA-dependent defense responses.

## Supplementary information

Supplementary information.
