# Supplementary material for: Reynoutria sachalinensis extract elicits SA-dependent defense responses in courgette genotypes against powdery mildew caused by Podosphaera xanthii
Source: Sci Rep. 2020 Feb 25;10:3354. doi: 10.1038/s41598-020-60148-6 (PMC7042220; doi:10.1038/s41598-020-60148-6)
Supplement: Supplementary file 1 — Supplementary information. [file 41598_2020_60148_MOESM1_ESM.pdf]

***Reynoutria sachalinensis* extract elicits SA-dependent defense responses in courgette genotypes against powdery mildew caused by *Podosphaera xanthii***

Theoni Margaritopoulou, Eleftheria Toufexi, Dimosthenis Kizis, George Balayiannis, Christos Anagnostopoulos, Andreas Theocharis, Leonidas Rempelos, Yerasimos Troyanos, Carlo Leifert, Emilia Markellou

Supplementary Information

Supplementary Fig. 1

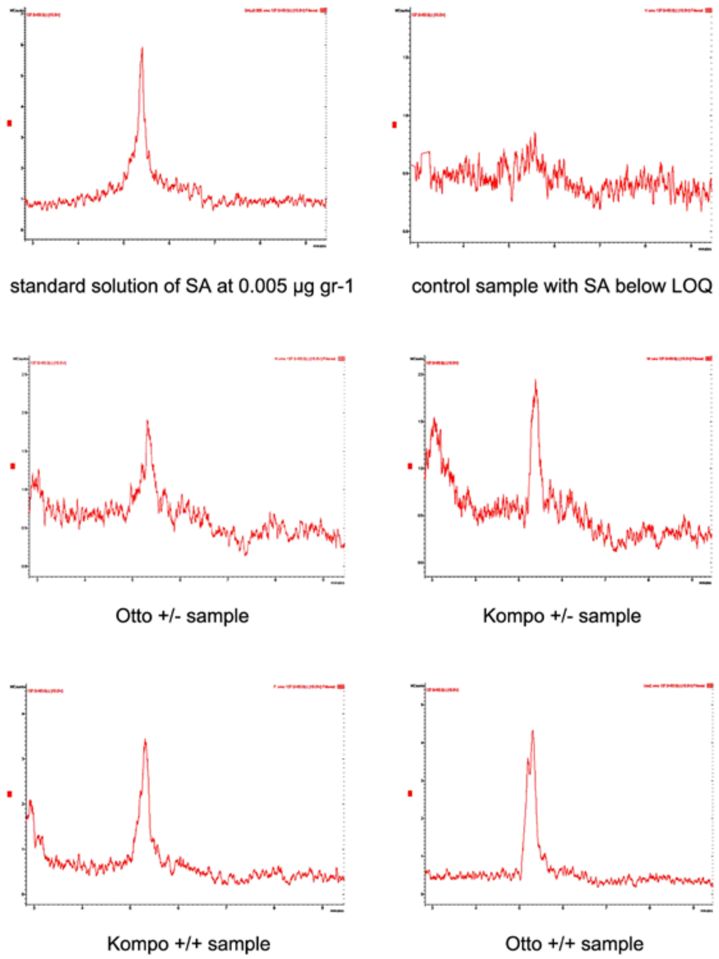


**Supplementary Fig. 1** Typical Selected Reaction Monitoring (SRM) chromatograms for all analytes used in this study and representative samples.

Table S1. List with the primers used in this study

| primer | sequence |
|---|---|
| CP-Ef1a-F | 5’-CCCGGACATCGTGACTTTAT-3’ |
| CP-Ef1a-R | 5’-ACCAGCTTCAAAACCACCAG-3’ |
| CP-PR1-F | 5’-AAACTCGGTGAGAATTGGATG-3’ |
| CP-PR1-R | 5’-CTTTGGCCTATGATATTGCCAC-3’ |
| CP-PAL-F | 5’-CATGGACAACACTCGTCTGG-3’ |
| CP-PAL-R | 5’-TTTGAAGGTAGCCCGTTGTT-3’ |
| CP-NPR1-F | 5’-GCCCAAGTGTTCAGAACCAAA-3’ |
| CP-NPR1-R | 5’-TCCAGTTCTTCCGAGCATTTC-3’ |
| CP-PR2-F | 5’-TGGTTCACCCCTTCTTGCTA-3’ |
| CP-PR2-R | 5’-ACAAGAGTTGGCGATTGCAT-3’ |
| CP-MYC2-F | 5’-GATCTGATGATCCAACAAGCC-3’ |
| CP-MYC2-R | 5’-ACACAGTGAAGGAGACAAGG-3’ |
| CP-VSP2-F | 5’-CTGATGAGTTTGATAGCTGGG-3’ |
| CP-VSP2-R | 5’-AGCAAAACCACCTTGAATCC-3’ |
| CP-ABF1-F | 5’-TAAGCACGGGAGGTGTAAC-3’ |
| CP-ABF1-R | 5’-CCGACCAAACGAATATGGAAC-3’ |
| CP-ETR1-F | 5’-CCATACGCATACGCGAAAAG-3’ |
| CP-ETR1-R | 5’-AGTATTAGCCCGTCCATGCC-3’ |
| CP-PDF1.2-F | 5’-GCCATGCTCAATTTCCTGG-3’ |
| CP-PDF1.2-R | 5’-CGTTTCAAACACACGTAGTAAG-3’ |
| CP-ERF1-F | 5’-GAACAAAGAGAGGGATTTGAGG -3’ |
| CP-ERF1-R | 5’-ATTTAGTAAGAACAGGGGATGC-3’ |
